# Understanding the Cellular and Molecular Mechanisms That Control Early Cell Fate Decisions During Appendicular Skeletogenesis

**DOI:** 10.3389/fgene.2019.00977

**Published:** 2019-10-11

**Authors:** Jessica Cristina Marín-Llera, David Garciadiego-Cázares, Jesús Chimal-Monroy

**Affiliations:** ^1^Instituto de Investigaciones Biomédicas, Universidad Nacional Autónoma de México, Ciudad Universitaria, Mexico City, Mexico; ^2^Instituto Nacional de Rehabilitación, Secretaría de Salud (SSA), Mexico City, Mexico

**Keywords:** skeletal stem cells, chondrogenesis, endochondral bone development, limb development, joint development

## Abstract

The formation of the vertebrate skeleton is orchestrated in time and space by a number of gene regulatory networks that specify and position all skeletal tissues. During embryonic development, bones have two distinct origins: bone tissue differentiates directly from mesenchymal progenitors, whereas most long bones arise from cartilaginous templates through a process known as endochondral ossification. Before endochondral bone development takes place, chondrocytes form a cartilage analgen that will be sequentially segmented to form joints; thus, in the cartilage template, either the cartilage maturation programme or the joint formation programme is activated. Once the cartilage differentiation programme starts, the growth plate begins to form. In contrast, when the joint formation programme is activated, a capsule begins to form that contains special articular cartilage and synovium to generate a functional joint. In this review, we will discuss the mechanisms controlling the earliest molecular events that regulate cell fate during skeletogenesis in long bones. We will explore the initial processes that lead to the recruitment of mesenchymal stem/progenitor cells, the commitment of chondrocyte lineages, and the formation of skeletal elements during morphogenesis. Thereafter, we will review the process of joint specification and joint morphogenesis. We will discuss the links between transcription factor activity, cell–cell interactions, cell–extracellular matrix interactions, growth factor signalling, and other molecular interactions that control mesenchymal stem/progenitor cell fate during embryonic skeletogenesis.

## Introduction

The formation of the vertebrate skeletal system is a paradigmatic model process for the study of differentiation, patterning, and morphogenesis during embryogenesis. The appearance of an endoskeleton during evolution has favoured the radiation of many forms of tetrapods that have adapted to many ecological niches (Vogel et al., 1996; Ohuchi et al., 1997; Shubin et al., 1997). An intricate network of genetic regulatory programmes that coordinate three-dimensional organization, differentiation, patterning, and morphogenesis hierarchically regulates skeleton formation during embryogenesis. During skeletogenesis, the sequences of events that occur during bone and cartilage development are temporally and spatially regulated to not only control the patterning of the early skeleton but also ensure the correct shapes of all skeletal elements. At the same time as patterning, precursor cells are committed to the cartilage lineage under precise spatial and temporal control of cell differentiation. Once chondrogenesis starts in the early stages of embryogenesis, cartilage forms and is gradually replaced by bone; however, it persists on articular surfaces and forms the sole skeletal support system for the larynx, trachea, bronchi, and other structures. Cartilage is usually an avascular tissue, except in areas where blood vessels pass through other tissues and in regions where endochondral ossification occurs (Mackie et al., 2008). According to its nature and visibility, cartilage can be subdivided into three varieties: 1) hyaline, 2) elastic, and 3) fibrous, with hyaline cartilage being the most widely distributed type. As a tissue, cartilage has multiple embryological origins, including the cranial neural crest (CNC) (Chai et al., 2000), dorsal paraxial mesoderm, and lateral plate mesoderm (LPM) (Hall, 1981; Takahashi et al., 2001). CNC cells migrate from the dorsal neural tube into the branchial arches and other regions of the developing face and head, where they give rise to bones and cartilage (Hall, 1981; Takahashi et al., 2001). The ribs and vertebrae originate from somites, which are formed by segmentation of the paraxial mesoderm. The somites de-epithelize ventrally to give rise to the sclerotome. Mesenchymal cells from the sclerotome receive signals from the notochord to form vertebrae and intervertebral discs, while cells from the lateral sclerotome migrate out to form the ribs (Chal and Pourquié, 2017). Finally, cells from the LPM participate in the establishment of the appendicular skeleton. LPM cells proliferate and migrate from the flanks of the embryo and form the limb buds (Vogel et al., 1996; Ohuchi et al., 1997; Jin et al., 2018). As development progresses, these initially undifferentiated cells interpret signals according to their positions and differentiate into a variety of tissues that compose the adult limb while simultaneously shaping each limb into its final form.

The aim of this review is to explore the earliest molecular processes that control cell fate decisions during appendicular skeletogenesis. Specifically, we will focus on the cellular and molecular mechanisms that govern the early fate decisions of mesenchymal stem/progenitor cells that give rise to the appendicular skeleton, using limb development as a model. Here, the term stem/progenitor cells will be used despite there is no evidence that mesenchymal cells are either stem or progenitor cells. Stem cells are considered to have the capacity to reproduce themselves. By asymmetric division, these cells can generate progeny with the ability to acquire distinct cell fates and to differentiate into functional cell types. Stem cells are present in many tissues for long durations and are regulated by the microenvironment, the stem cell niche. In contrast, progenitor cells arise from asymmetric division of stem cells and differentiate into distinct functional cell types. When progenitor cells proliferate to amplify their populations, they are also called transient amplifying cells (Slack, 2018). First, we will review the initial process that leads to the recruitment of mesenchymal stem/progenitor cells to the chondrocyte and osteoblast lineage and to the formation of skeletal elements located in the core of the limb and the subsequent process of endochondral ossification in long skeletal elements. We will also explore the early steps of joint specification.

### A Brief Overview of Limb Development

**Figure 1:**
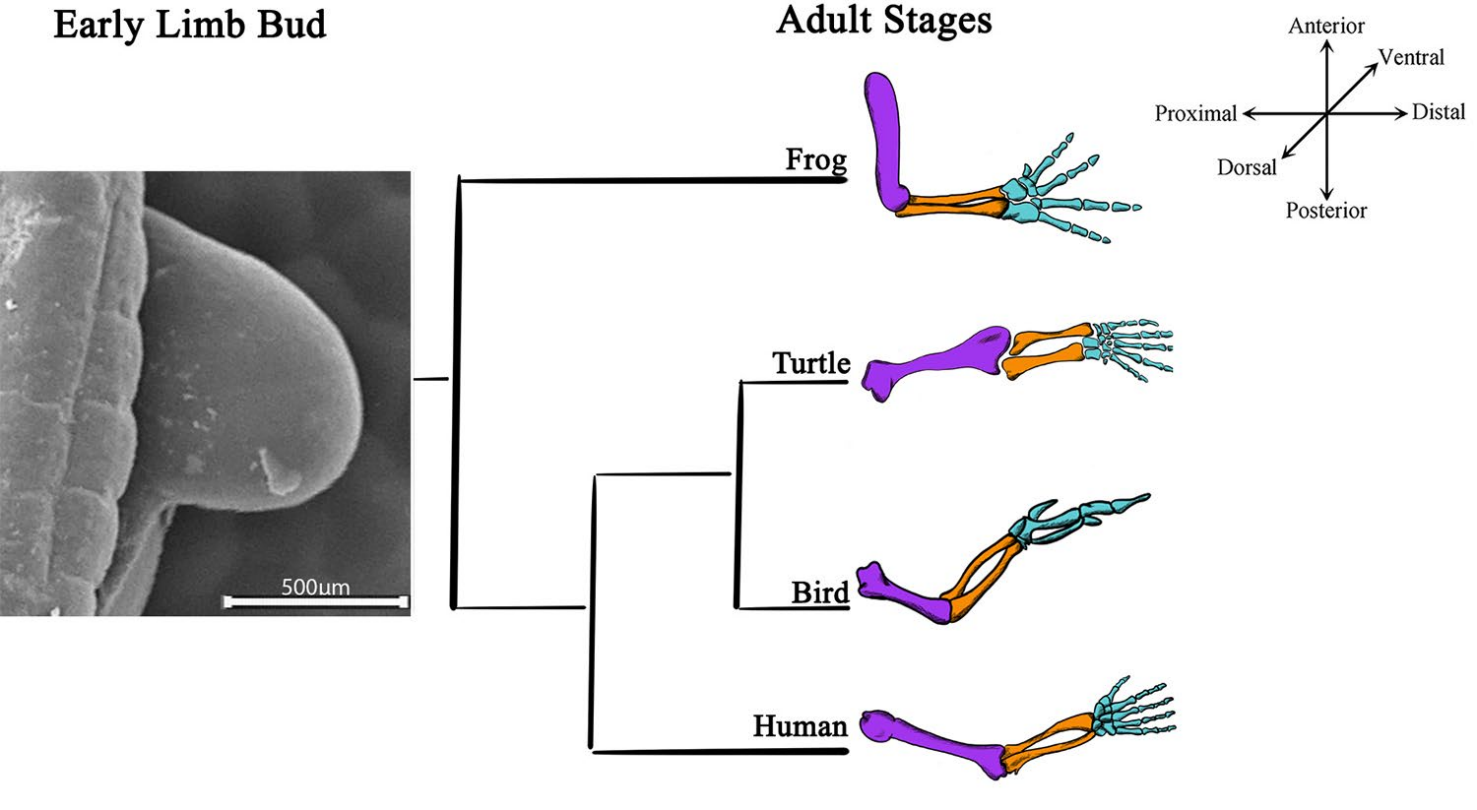
Limb bud development and adult limb structures are conserved among tetrapods. A scanning electron microscopy image of a 22HH chick forelimb shows limb bud, the structure where all the skeletal elements arise during development. Schematic representations of adult skeletal structures of forelimbs from distinct tetrapods (frog, turtle, bird, and human) are shown. The most proximal skeletal element, the stylopod, is characterized for a single skeletal element (purple); two middle elements represent the zeugopod (orange), and the distal region highly segmented represents the autopod (blue).

Tetrapod limbs are adapted to different habitats. Despite their diversity, limbs have three anatomical regions that can be distinguished: the stylopod in the proximal region, which gives rise to the humerus of the forelimb and the femur of the hindlimb; the zeugopod, which corresponds to the two middle elements, the radius/ulna of the forelimb or the tibia/fibula of the hindlimb; and the autopod, which corresponds to the highly segmented distal elements, developing into the metacarpal/carpal/finger bones of the forelimb and the metatarsal/tarsal/toe bones of the hindlimb (**Figure 1**; Towers and Tickle, 2009; Tickle, 2015).

**Figure 2:**
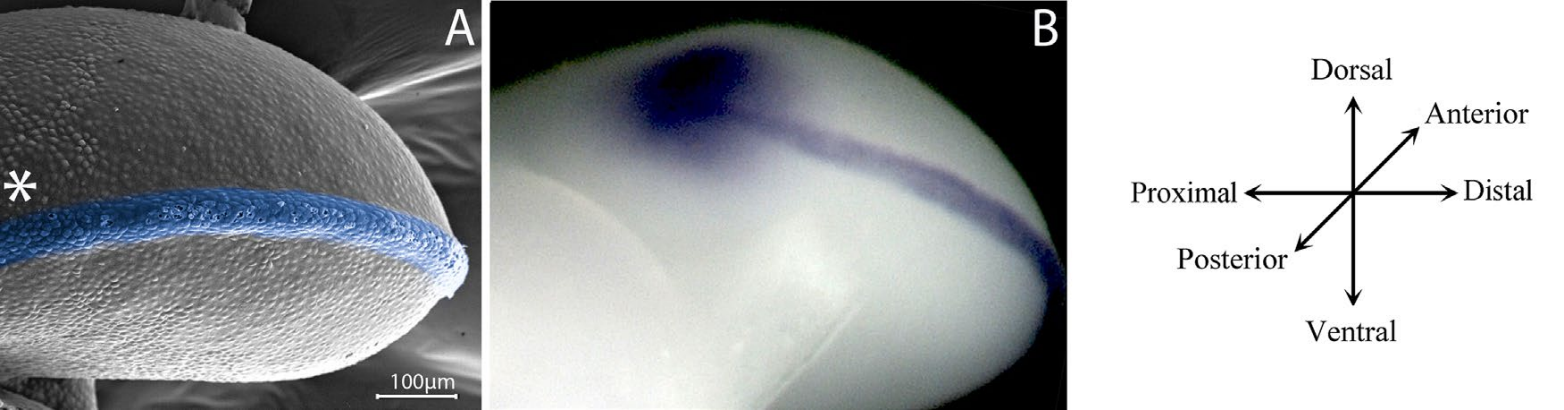
Signalling centres controls limb development. **(A)** A posterior view of a scanning electron microscopy of a 22HH chicken hindlimb bud showing in blue the apical ectodermal ridge (AER) between the dorsal and ventral ectoderm. The localization of the zone of polarizing activity (ZPA) is showed with an asterisk. **(B)** In situ hybridization in a 22HH chicken hindlimb showing the expression of Shh in the ZPA and Fgf8 in the AER, signalling centres of the limb bud.

The beginning of limb development is evidenced by the emergence of a limb bud (Tickle, 2015). Embryonic limb buds are formed by mesenchymal cells covered with ectoderm. The mesenchyme gives rise to the skeleton and ligaments, while the ectoderm gives rise to the skin and its derivates. However, although a homogeneous mesenchymal cell population is initially observed, cell populations that secrete molecular signals to establish limb spatial patterns are localized in certain limb bud zones (Shimizu et al., 2007; Zeller et al., 2009; Tickle, 2015) i.e., the arrangement of limb structures and the formation of a functional limb originates from the spatiotemporal organization of cells. The establishment of spatial patterns is closely related to morphogenesis, the process by which an organism develops its final shape. The three-dimensional organization of the limb depends on molecular interactions between three different signalling centres that govern limb shape and patterning in the proximo-distal (PD), antero-posterior (AP), and dorso-ventral (DV) axes (Towers and Tickle, 2009; Tickle, 2015). The outgrowth of the limb bud is coordinated by the apical ectodermal ridge (AER), positioned at the distal part of the limb at the boundary between the dorsal and ventral ectoderm (**Figure 2**; Saunders, 1948; Laufer et al., 1997; Rodriguez-Esteban et al., 1997). This structure releases signals to the undifferentiated region beneath it, promoting proliferation and maintaining the undifferentiated state of mesenchymal stem/precursor cells. Once mesenchymal stem/progenitor cells leave the region under the influence of the AER, they acquire spatiotemporal cues that allow them to commit to different cell lineages (Tabin and Wolpert, 2007). On the other hand, the AP polarity of the limb is directed by the zone of polarizing activity situated in the posterior region of the early limb (Saunders and Gasseling, 1968; Riddle et al., 1993). Finally, the dorsal ectoderm controls the DV polarity of the limb (**Figure 2**; Yang and Niswander, 1995, Parr and McMahon, 1995). Mesodermal cells from all axes interpret the molecular signals from the three signalling centres, giving rise to skeletal elements, muscles, tendons, and ligaments.

In all vertebrates, the formation of the appendicular skeleton originates from a cartilage template initiated in the core of the limb mesenchyme (Shum et al., 2003). The development of appendicular skeletal elements requires the proliferation and migration of mesenchymal cells from the LPM to the limb bud (Shimizu et al., 2007). Mesenchymal cells at the periphery of the limb bud are maintained in an undifferentiated state by signals released from the ectoderm (ten Berge et al., 2008). However, in the centre of the limb bud, chondrogenic signals induce the aggregation of mesenchymal stem/progenitor cells, giving rise to condensations that will later form the cartilage templates. The production of cartilage anlagen of specific sizes, shapes, and positions allows the precise patterning of limb skeletal elements. Except in the joints, the cartilage tissue is eventually replaced by bone in a process called endochondral ossification.

### Maintenance of the Undifferentiated State of Limb Mesenchyme Prior to Skeletal Specification

Multiple cell types form an adult limb, but cartilage, bone, perichondrium, dermis, ligaments, and tendons originate from the limb mesenchymal stem/progenitor cells. Preceding lineage commitment, mesenchymal stem/progenitor cells are maintained in an undifferentiated state due to the influence of the AER and ectodermal signals. When they abandon the area of influence of these signals, begin the cellular commitment to give rise to different cellular lineages.

#### The Importance of the Establishment of the AER-FGF Signalling

**Figure 3:**
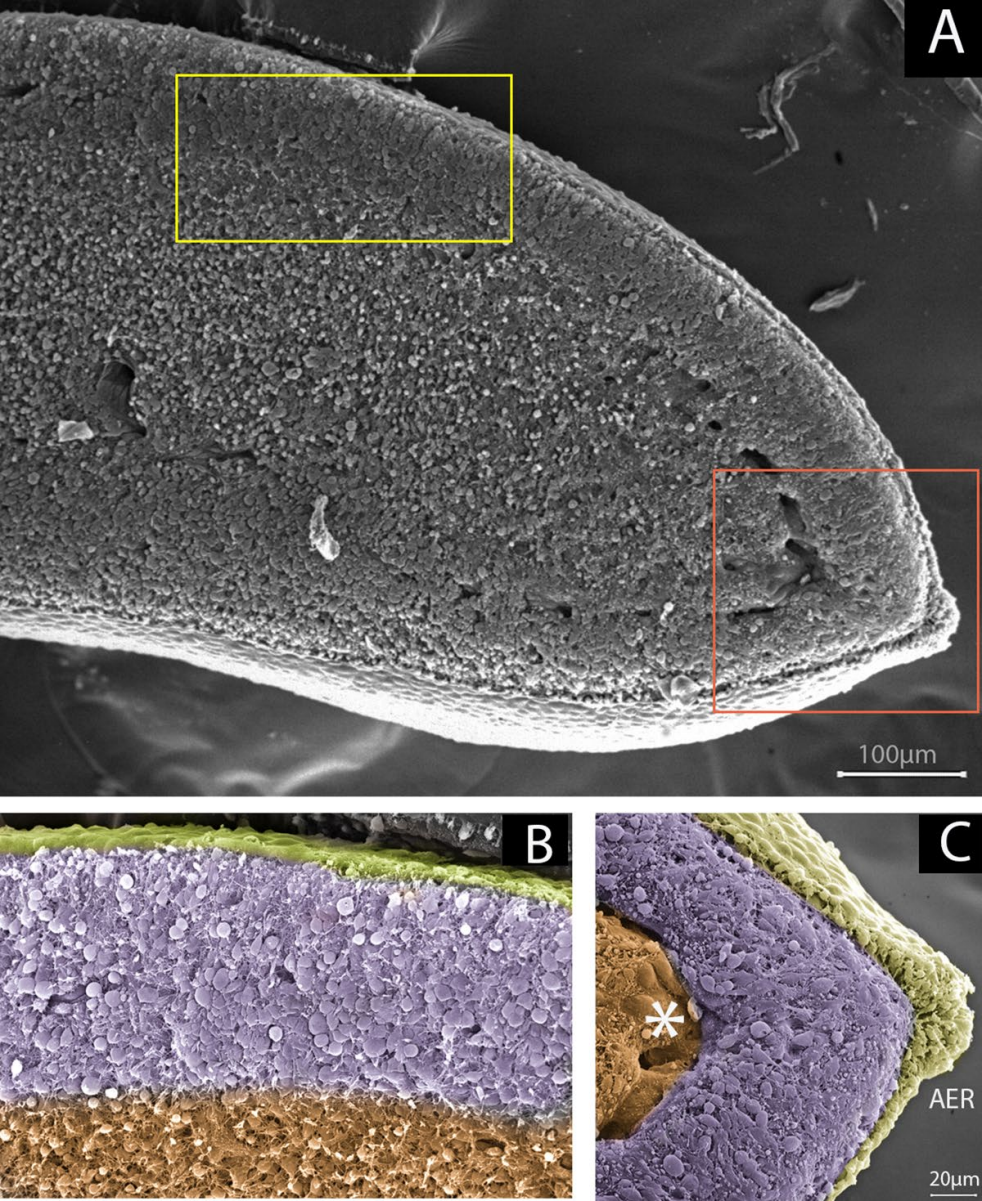
Undifferentiated zones beneath limb ectoderm as reservoirs of stem/progenitor cells. **(A)** Scanning electron microscopy of a sagittal section of a 23HH chicken forelimb showing the undifferentiated zone. Yellow and orange squares-marked regions represents the image showed in B and C respectively. **(B)** Magnification of yellow boxed area. The undifferentiated zone, in purple, is under the dorsal ectoderm (yellow) influence. The region where mesenchymal cells are committed to chondrogenic/tenogenic lineage is showed in orange. **(C)** Magnification of orange boxed area. The undifferentiated zone (purple) underlies the Apical Ectodermal Ridge (AER) and dorsal and ventral ectoderm (yellow). Notice that the marginal vein, indicated with an asterisk, delimits the undifferentiated zone and the committed zone (orange).

The undifferentiated zone under the AER is approximately 200 µm in size and is located in the distal region of the limb bud (**Figure 3**; Summerbell et al., 1973; Dudley et al., 2002). Pioneering work by Saunders (Saunders, 1948) and later work by Summerbell (Summerbell, 1974) demonstrated the role of the AER in the outgrowth and formation of the skeletal elements of the embryonic limb. Using chicken embryos, they showed that AER removal at progressive stages of limb development caused a progressive loss of distal elements of the limb (Saunders, 1948; Summerbell, 1974). In the chicken embryo, the AER becomes distinguishable at Hamburger–Hamilton stage 18 (Hamburger and Hamilton, 1992) (18HH) during development, when distal ectodermal cells acquire columnar shapes distinguishing them from the cuboidal ectoderm. At stage 20HH, the AER becomes a pseudostratified epithelium that is maintained until the 23–24HH stage (Todt and Fallon, 1984). On the other hand, AER initiation in the mouse forelimb starts at approximately embryonic day 9 (E9). It is known that Wnt3a/β-catenin signalling in chicken and Wnt3/β-catenin signalling in mouse is required to maintain the AER after its initiation and maturation (Barrow et al., 2003; Kengaku et al., 1998). In 2002, it was demonstrated that both fibroblast growth factor (FGF) 8 and 4 signalling from the AER play instructive roles and are essential for the survival of mesodermal cells, maintaining the viability of the underlying AER mesodermal cells to ensure sufficient availability of progenitors for normal formation of the skeletal elements (Sun et al., 2002). However, *Fgf9* and *Fgf17* are also expressed in the AER and contribute to limb development. Interestingly, an *Fgf8* knockout (KO) mutant has been found to show more severe limb defects than individual and compound *Fgf4/Fgf9/Fgf17* mutants. This result suggests that the presence of *Fgf8* is sufficient for normal limb development. An explanation for the diverse range of phenotypes obtained with various *Fgf* KOs is that the AER-FGFs (*Fgf8, Fgf4, Fgf9*, and *Fgf17*) are functionally equivalent but differ in the extent to which they contribute to the AER-FGF signal; thus, changes in the different components produce different changes in the total AER-FGF signal (Mariani et al., 2008). On the other hand, Lu et al. (2008) through genetic ablation of FGFR2 function found evidence suggesting that FGFR2 promotes the survival of AER cells and interacts with Wnt/β-catenin signalling during AER maintenance. Additionally, using *Hoxa13* as an early autopod progenitor cell marker, the authors found that premature AER loss in mutant limb buds may delay the generation of autopod progenitors, in turn preventing the progenitors from reaching the threshold number required to form a normal (Lu et al., 2008). However, mesenchymal expression of *Fgfr2* (Coumoul et al., 2005) and *Fgfr1* (Li et al., 2005; Verheyden et al., 2005) is also necessary for skeletal progenitor cells to respond to AER signals.

#### Molecular Control in the Maintenance of an Undifferentiated State of Mesodermal Cells

In the first stages, all mesenchyme in the limb bud is composed of undifferentiated cells. While the limb grows, an undifferentiated distal zone is always maintained (**Figure 3**). The region from which digital rays later extend and where joints are sequentially formed also features an undifferentiated zone known as the digital crescent (DC) (Montero et al., 2008) or phalanx-forming region (PFR) (Suzuki et al., 2008), which is positive for pSMAD1/5/8 and pSMAD2. The maintenance of mesenchymal stem/progenitor cells during development in an undifferentiated, proliferative, and viable state is highly regulated by ectodermal signals.

It is known that combinations of Wnt and FGF signals from the limb ectoderm, specifically FGF8 and WNT3A signals, have different effects on the mesenchymal stem/progenitor cells of the undifferentiated region than either signal alone. Mesenchymal cells are maintained in a multipotent and proliferative state by the synergistic action of both growth factors, but they retain the ability to undergo chondrogenesis. In the absence of both signals, mesenchymal stem/progenitor cells exit the cell cycle and begin chondrogenic differentiation. Continuous exposure to Wnt induces *Nbl1* expression, which maintains proliferation and re-specifies the cells towards soft connective tissue lineages (ten Berge et al., 2008). Additionally, *N-Myc* plays a significant role in the expansion of undifferentiated mesenchymal cells, which gives rise to chondrocyte and osteoblast progenitors, while *c-Myc* participates in the proliferative expansion of osteoblast progenitors (Zhou et al., 2011). Furthermore, FGF and WNT family members secreted from the ectoderm promote *N-Myc* expression in the mouse limb and consequent proliferation of the underlying mesenchyme lineages (ten Berge et al., 2008). Thus, newly generated undifferentiated cells cannot reach the most central part of the limb bud and are maintained in their undifferentiated state. When undifferentiated mesenchymal cells are far enough from the AER signals, they exit the cell cycle and commit to becoming *Sox9*-positive chondro-osteoprogenitors (Akiyama et al., 2005). In addition, in the chick embryo has been demonstrated that the dorsal ectoderm releases a probable chondrogenesis inhibitory factor (WNT6), which restricts the formation of cartilage towards the central region of the limb. If the dorsal limb ectoderm is removed, chondrogenesis differentiation extends to the mesenchyme under the dorsal ectoderm, probably at the expense of tendon formation (Geetha-Loganathan et al., 2010).

Thus, in the appendicular skeleton, the commitment of mesenchymal cells to the chondrogenic and tenogenic lineages occurs when cells in the undifferentiated zone reach the differentiation front and leave the influence of the AER and ectodermal signals (Tabin and Wolpert, 2007). The details of the molecular basis of the maintenance and cell fate regulation of mesenchymal stem/progenitor cells in the limb have been elucidated. Kumar and Lassar demonstrated in mouse embryonic limb that Wnt signals irreversibly block the induction of chondrogenesis through CpG methylation and H3K27me3 modification in the *Sox9* promoter. In contrast, the FGF signal blocks the recruitment of the *de novo* methyltransferase DNMT3A to the *Sox9* promoter, maintaining competence for eventual *Sox9* gene expression by blocking Wnt signals from inducing stable CpG methylation at the *Sox9* locus. Thus, FGF signalling controls whether *Sox9* expression is irreversibly or reversibly silenced by Wnt signals in limb bud undifferentiated mesenchymal cells (Kumar and Lassar, 2014). Furthermore, the arginine methyltransferase PMRT5 present in the DC/PFR is considered to promote the survival and proliferation of mesenchymal stem/progenitor cells, thus maintaining the undifferentiated pool of progenitor cells and limiting cell differentiation in the distal tips of digital rays (Norrie et al., 2016). Moreover, the Notch pathway is active in mesenchymal stem/progenitor cells and inhibits their differentiation *via* upregulation of the transcription factor *Twist1* (Tian et al., 2015).

On the other hand, in chicks and mice, hypoxic environments and hypoxia-inducible factor 1 alpha (*Hif1a*) upregulation participate in the fate determination that initiates the Sox9-positive chondrocyte lineage and represses the *Runx2*-positive osteoblast lineage and tenogenic differentiation (Salim et al., 2004; Amarilio et al., 2007; Lorda-Diez et al., 2013). Furthermore, the initial low-level *c-Myc* expression in newly *Sox9*-positive cells has been proposed to promote the proliferation of these cells but maintain their multipotent progenitor character (Zhou et al., 2011). Ultimately, limb mesenchymal cells are definitively committed to cartilage or soft connective tissue lineages at the chick embryo 24HH stage (Searls and Janners, 1969).

#### Coordinated Regionalization and Differentiation of Limb Mesodermal Cells

It is known that limb patterning and cell differentiation occur in an orchestrated manner (Dudley et al., 2002; Sun et al., 2002). A detailed fate map for the limb bud of the chicken at stage 19HH demonstrates that only the distal undifferentiated mesenchyme is maintained in a non-regionalized condition and that each limb structure is likely to be regionalized in the proximal-to-distal direction (Sato et al., 2007). Additionally, using KO mutant mice lacking different combinations of *Fgf4*, *Fgf8*, *Fgf9*, and *Fgf17*, it was demonstrated that AER-FGFs function as initial distal signals during the early stages of limb development, playing instructive roles in specifying distal domains for PD patterning and repressing *Meis1* expression (Mariani et al., 2008). Thus, the co-existence of two parallel mechanisms during limb PD patterning has been demonstrated: one based on MEIS, retinoic acid (RA), and FGF signalling and a second based on chromatin regulation that delays *Hoxa13* activation to provide the time needed for the accumulation of sufficient precursors to ensure proper limb formation (Rosello-Diez et al., 2014). Hence, cells in the undifferentiated zone are constantly under the influence of FGF signalling. Once undifferentiated cells cross the differentiation front, aside from committing to a chondrogenic lineage, they become sequentially specified as stylopod, zeugopod, and autopod cells due to increased RA signalling (Rosello-Diez et al., 2014). On that basis, undifferentiated cells interpret signals that lead them to differentiate while they acquire their identities from a specific segment of the limb.

#### Are Early Limb Mesodermal Cells Stem Cells?

The existence of a distal undifferentiated zone in the limb as a source of stem/progenitor cells has been well established. It has been clearly demonstrated *in vitro* and *in vivo* that mesenchymal cells at early stages of development are capable of differentiating into different limb lineages (Ahrens et al., 1977; Akiyama et al., 2005; Marin-Llera and Chimal-Monroy, 2018), Even extra-limb lineages belonging to ectodermal and endodermal germ layers have been obtained *in vitro* (Jiao et al., 2012). However, it remains unclear whether the mesenchyme is composed of a collection of distinct types of stem/progenitor cells with restricted differentiation potential or of a group of equipotential cells capable of giving rise to the same cell lineages. Using a library of retroviral vectors to trace the cellular fate and location of the progeny of individually marked single cells during limb development, Pearse II and collaborators demonstrated that subsets of multipotent cells that can give rise to between two and five different lineages (muscle connective tissue, tendon, dermis, perichondrium, and cartilage) are present in the limb mesenchyme. Their results showed that at the 19HH stage, cells are not specified to any lineage, and at least a subset of progenitor cells can generate five mature tissue types. Additionally, a progressive restriction of multipotency was observed through development. In that work, the authors concluded that cells in the early limb bud are not committed to individual fates, nor do they appear to be restricted to any particular PD segment along the length of the limb bud (Pearse et al., 2007). Although the multipotency of undifferentiated limb cells is clear, the signature of each type of subpopulation has not been determined, nor has it been determined whether the cells are multipotent stem cells. Detection of specific signatures of cell surface molecules, such as mesenchymal stromal cell-associated markers (MSCams), can enable the identification and study of distinct limb subpopulations at different developmental stages. In this sense, it has been demonstrated that few limb cells express different MSCams (Marin-Llera and Chimal-Monroy, 2018). Gretel Nusspaumer et al. traced the ontogeny and relationships of distinct mesenchymal stromal cell populations previously reported in the mouse limb and found that the PDGFRa^pos^/CD51 cell population is the largest population of progenitors, containing mouse skeletal stem cell (mSSC) (CD200^pos^, CD51^pos^, CD90^neg^, CD105^neg^, and 6C3^neg^) and PαS subpopulations. Additionally, in the PαS population (PDGFRa^pos^, Sca1^pos^, CD45^neg^, and TER119^neg^), four subpopulations can be distinguished based on their potential to differentiate into cartilage (Nusspaumer et al., 2017). The use of mesenchymal stromal cell (MSC) surface markers enables the identification, isolation, and characterization of different types of cells in the limb; however, the expression of these molecules alone does not indicate stemness. In particular, when expression has been measured after culture, it has been demonstrated that cells from a variety of tissues acquire surface signatures or modify their surface signatures *in vitro* (Qian et al., 2012; Lei et al., 2013; Guimaraes-Camboa et al., 2017; Marin-Llera and Chimal-Monroy, 2018).

Despite the existing knowledge of the origins, maintenance, and cell fate regulation of stem/progenitor cells in the limb, their self-renewal capacity and the timing of cell fate decisions by undifferentiated cells in the context of the developing limb is still unclear.

### Cartilage Commitment and Differentiation

Some of the important characteristics of skeletal development are that all skeletal elements are confined to specific locations with specific shapes and are present in defined numbers. The particular organization of the skeleton depends on its particular locomotor function for each species. However, the initial steps that drive chondrogenesis are conserved in many species of vertebrates.

**Figure 4:**
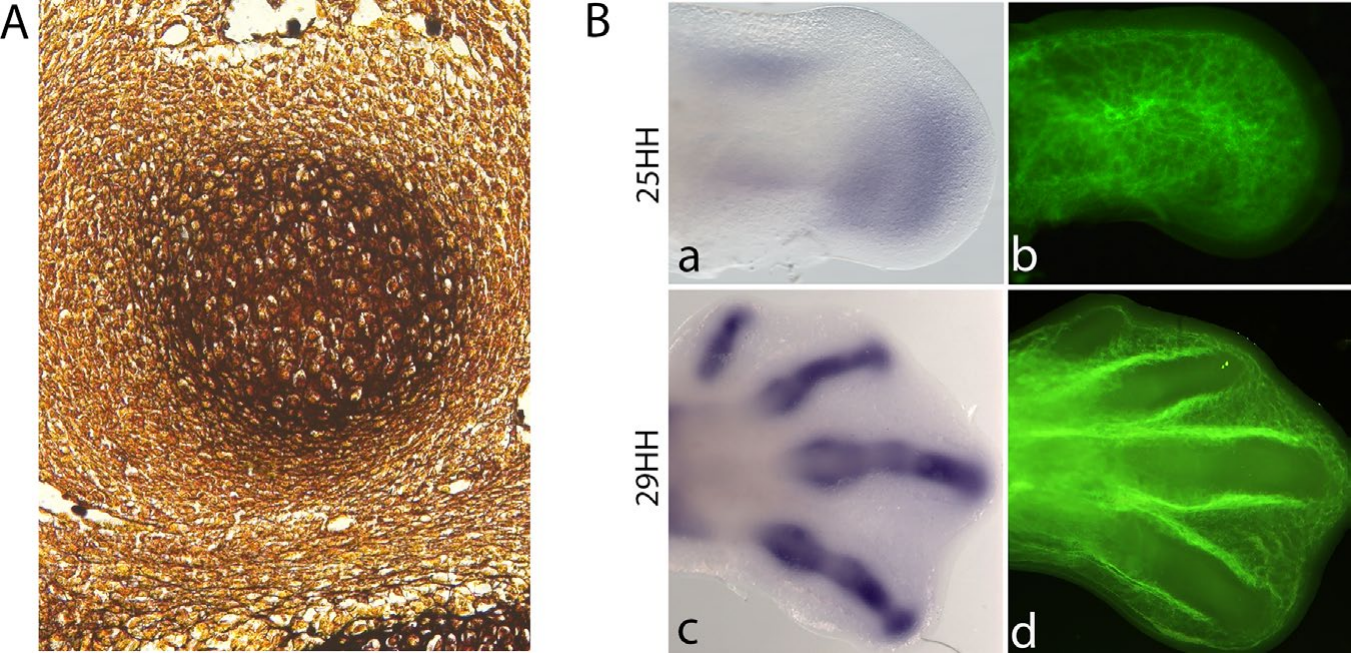
Chondrogenesis starts when the limb mesenchymal cells leave the influence of the AER-FGFs signalling. **(A)**. Histological section of a silver-stained 28-HH chicken digit showing a cartilage condensation. **(B)**. In situ hybridization for Sox9 (a,c) and blood vessel cell-tracker staining (b,d) in chicken hindlimbs at 25HH and 29HH stages. Noticed that cartilage differentiation is accompanied by vascular regression. Avascular zones can be observed at the later stage in the digits.

Stem/precursor cells are committed to the chondrocyte lineage in the core of the limb. Ectoderm tissue releases signals that block cartilage differentiation in the margin of the early limb bud, restricting cartilage formation to the core of the limb mesenchyme (ten Berge et al., 2008; Cooper et al., 2011). Once chondrogenic signals are received and/or inhibitory signals are removed, mesenchymal cells begin to aggregate and differentiate into early chondrocytes, building the cartilage anlagen, proliferating and producing characteristic extracellular matrix (ECM; **Figure 4A**; Hall and Miyake, 1995). During this process, Wnt and FGF signals from the ectoderm control the expression of Sox9 through epigenetic modification (Kumar and Lassar, 2014).

#### First Steps of Chondrocyte and Osteoblast Commitment

As mentioned above, AER-FGF signals determine whether mesenchymal cells are responsive to chondrogenic signals by regulating epigenetic modifications in the *Sox9* promoter (Kumar and Lassar, 2014). However, once *Sox9* is expressed, there is evidence that functional and physical interactions occur between Sox9 and β-catenin during cartilage development. If an interaction occurs between the Sox9 C-terminus and β-catenin, the transcriptional activity of the β-catenin/Tcf-Lef complex is inhibited (Akiyama et al., 2004; Topol et al., 2009). Alternatively, translocation of the Sox9 and β-catenin complex into the nucleus is necessary and sufficient to induce β-catenin degradation (Topol et al., 2009).

The cellular recruitment of mesenchymal cells towards the chondrogenic lineage occurs in the undifferentiated zone beneath the AER (**Figure 4B**). The formation of precartilage condensations in the core of the limb depends on cell–cell and cell–ECM interactions. N-Cadherin and N-CAM are two cell adhesion molecules expressed during precartilage condensation formation. Blocking the function of these molecules prevents cell aggregation and subsequent cartilage differentiation in micromass cultures prepared from chicken limb precartilage cells (Chuong et al., 1993; Widelitz et al., 1993; Oberlender and Tuan, 1994a, Oberlender and Tuan, 1994b). However, no limb phenotype has been reported in N-CAM knockout mice (Cremer et al., 1994). Regarding cell–ECM interactions, fibronectin and tenascin are ECM proteins expressed during the formation of precartilage condensations (Hall and Miyake, 1995). Similarly, blocking the function of fibronectin with a specific antibody for exon IIIA of fibronectin (Gehris et al., 1997) or with an antibody directed against the 29 kDa amino-terminal heparin-binding domain of fibronectin and the oligopeptide gly-arg-gly impedes the formation of pre-cartilage condensations (Frenz et al., 1989). Interestingly, blocking the function of β1 integrins inhibits cell aggregation and thus impedes cartilage differentiation, at least *in vitro* (Shakibaei, 1998).

However, the condensing mesenchyme in the limb bud shows a bi-potential character, as it is able to differentiate into both chondrogenic and osteogenic lineages. As mentioned above, the chondrogenic lineage is characterized by the expression of Sox9, whereas the osteogenic lineage is characterized by the expression of Runx2. The activation of Wnt/β-catenin signalling is involved in controlling the decision to become osteoblasts or chondrocytes in non-committed cells in the undifferentiated region (Hill et al., 2005). Conditional deletion of β-catenin in the limb mesenchyme results in ectopic cartilage differentiation at the expense of osteoblast differentiation (Akiyama et al., 2005; Day et al., 2005; Hill et al., 2005). Similarly, inhibition of Runx2 results in the inhibition of osteogenesis but not chondrogenesis. Thus, during direct ossification processes, such as intramembranous ossification, Wnt/β-catenin signalling is active; *Sox9* expression is downregulated; and *Runx2* expression is activated. In contrast to Runx2, deletion of the *Sox9* gene before chondrocyte commitment results in the complete inhibition of skeletal element formation (Akiyama et al., 2002). Thus, during the formation of long skeletal bones, Wnt/β-catenin signalling is inhibited such that *Sox9* expression is upregulated. In this context, condensing pre-cartilage cells exit the cell cycle and express Hif1a, and a hypoxic environment is created. At the same time, hypoxia inhibits osteogenic and tenogenic differentiation, but the chondrogenic lineage avoids this inhibition because of the upregulation of *Sox9* by Hif1α (Salim et al., 2004).

#### Chondrogenesis During Digit Formation

During digit formation in chicken embryos, chondrogenic differentiation begins when the TGFβ/activin signalling pathway directs the pool of mesenchymal precursor cells in the DC/PFR region to a cartilage fate by inducing *Sox9* and *Bmpr1b* expression (Montero et al., 2008). *Activin/Tgfβ* expression is restricted to the earliest chondrogenic condensations of the growing tips of digits until the last phalanx is formed but is not observed in cellular condensations of proximal skeletal elements. In contrast, *Activin* βB transcripts are observed only in the initial stages of digit formation. The expression of activin receptor II, *ActRIIb*, is intense in the precursor cells surrounding the distal tip of the digit. In addition, *Tgfβ2* expression is observed at stage 29 during the formation of growing digits (Merino et al., 1998). In contrast to chick embryos, single mouse mutants for *Tgfβ1, Tgfβ2,* and *Tgfβ3* do not exhibit digit abnormalities that suggest any participation of these genes in digit development. Nevertheless, severe syndactyly is observed in double knockout animals for *Tgfβ2* and *Tgfβ3* (Shull et al., 1992; Kaartinen et al., 1995; Dunker et al., 2002). Moreover, Activin- or Follistatin-deficient mice show no digit phenotype (Matzuk et al., 1995a; Matzuk et al., 1995b; Matzuk et al., 1995c).

During digit development, it has been shown that non-canonical Wnt signalling through Wnt5a occurs at higher levels than Wnt/β-catenin signalling in the distal tips of mouse limb buds. In *Wnt5a* *^-^* */* *^-^* animals, chondrocyte differentiation is constrained because the levels of β-catenin are elevated, inhibiting *Sox9* expression; however, inhibition of β-catenin partially rescues chondrocyte differentiation (Topol et al., 2003). These results suggest that Wnt5a signalling promotes cartilage differentiation by inhibiting the antagonistic effect of Wnt/β-catenin signalling (Topol et al., 2003). On the other hand, the antichondrogenic *Sox* trio (Sox4, Sox11, and Sox12) cooperates to stabilize the β-catenin protein and replace Sox9 in the Sox9-β-catenin complex (Bhattaram et al., 2014). The antichondrogenic Sox trio also inhibits the activity of GSK3β, preventing the formation of degradation complexes. Interestingly, Wnt antagonists such as *Frzb1* are expressed during chondrogenesis in skeletal elements (Wada et al., 1999), and misexpression of Frzb1 in the developing limbs of chick embryos maintains chondrocytes at the initial stages of differentiation; they do not begin chondrocyte maturation. Thus, the skeletal elements are short with no endochondral ossification or joint fusion (Enomoto-Iwamoto et al., 2002), suggesting that these antagonists protect cartilage from the inhibitory effects of Wnt/β-catenin signalling on *Sox9* expression. However, in Frzb-knockout mice, no defects in early skeletogenesis are observed (Lories et al., 2007).

It has been shown that TGFβ and activin can induce extra digit formation during development (Ganan et al., 1996). The experimental model of ectopic digit formation induced by activin/TGFβ in interdigital tissue in chicken embryos is an excellent chondrogenesis model with which to elucidate the early molecular cascade that leads to cartilage differentiation *in vivo* (Chimal-Monroy et al., 2003). This model shows that the expression of *Sox9* is induced immediately after TGFβ treatment and that the expression of *Smad6* and *Bambi* [bone morphogenetic protein (BMP) antagonists] is also inhibited, favouring the receptivity of precartilage cells to BMP/SMAD1/5/8 signalling. BMP signalling plays an important role in prechondrogenic condensation and chondrogenic differentiation during digit formation (Pizette and Niswander, 2000; Barna and Niswander, 2007). Although, it is thought that the onset of chondrogenesis depends on BMP signalling, double mutant mice for *Bmp2* and *Bmp4* display skeletal malformations in the stylopod and the zeugopod, and in this region, one skeletal element is missing, whereas in the autopod, the size of the skeletal elements is reduced, and the two posterior-most digits are absent. Thus, although some skeletal elements are lost in the absence of Bmp2 and Bmp4, chondrogenic differentiation continues normally in the other condensations that are formed. The skeletal defects present in double mutant mice for *Bmp2* and *Bmp7* are less severe than those observed in double mutant mice for *Bmp2* and *Bmp4*. However, skeletal differentiation is normal (Bandyopadhyay et al., 2006).

Treatment of digit primordia in the DC/PFR region with different BMP proteins promotes expansion of cartilage at the digit tips in the chick limb (Macias et al., 1997; Merino et al., 1999a). In contrast, treatment with BMP antagonists such as Noggin and Gremlin results in digit truncation (Merino et al., 1999b). These results suggest that BMP plays an important role in the control of digit formation at two levels, first promoting cartilage condensation and second promoting chondrogenic differentiation. Nevertheless in the experimental model of ectopic digit formation, TGFβ but not BMP treatment induces the molecular cascade of chondrogenesis, and *Bmpr1b* is induced after 6 h of TGFβ treatment. BMP treatment induces cell death in the interdigital tissue, while treatment with BMP antagonists inhibits cell death (Chimal-Monroy et al., 2003). However, if BMP protein is implanted after 6 h of TGFβ treatment, coinciding with the time of *Bmpr1b* expression, BMP enhances chondrogenesis, promoting cartilage condensation and *Sox9* expression (Chimal-Monroy et al., 2003). Thus, it is possible that during digit development, BMP may act as a permissive signal. In addition, it is known that BMP signalling promotes the compaction of prechondrogenic cells during cell condensation (Barna and Niswander, 2007). This compaction promotes cohesive cell behaviour in mesenchymal cells to delineate the boundaries and sizes of cartilage elements. During the formation of digit primordia, the expression of the Iroquois (*Irx*) genes *Irx1* and *Irx2* is detectable at the boundaries of skeletal condensations and non-cartilage tissue (Diaz-Hernandez et al., 2013). It has been suggested that this expression may reflect the range of diffusion of TGFβ at growing digits to promote chondrogenesis, and IRX proteins might allow the formation of the prospective perichondrium by repressing the expression of genes involved in the cell death process (Diaz-Hernandez et al., 2013). A similar function has been suggested for *SoxC* genes, which may specify the fates of perichondrium and joint cells through a β-catenin-independent mechanism (Bhattaram et al., 2014).

#### The Earliest Molecular Steps of Chondrogenic Specification

Recently, on the basis of the molecular cascade for chondrogenesis induced in the experimental model of ectopic digit formation, it was established that three consecutive periods precede cartilage formation (Lorda-Diez et al., 2011). The first corresponds to the first three hours and is a pre-condensation stage characterized by the upregulated expression of *Sox9, Scleraxis, Ephrin A5,* and *Bigh3*, which are important regulators of cell adhesion. The functions of these molecules, together with cell morphology controlled by the actin cytoskeleton, are very important for chondrogenesis, as demonstrated by the fact that co-treatment with cytochalasin D inhibits chondrogenesis induced by TGFβ. Interestingly, new early molecular markers, the matricellular proteins CCN1 and CCN2, have been discovered. They are expressed very early at the same time as *Sox9*, but they are unable to induce chondrogenesis; instead, they induce the expression of *Tenascin-C*. On this basis, the commitment of stem/precursor cells to the chondrocyte lineage occurs in this period. Thus we suggest that it should be named the commitment stage, as the master gene *Sox9* is expressed as early as 30 min, triggering gene regulatory networks that control the correct positioning of all skeletal elements in time and space. The second period is named the condensation stage, which corresponds to the period in which committed cells initiate the process of mesenchymal condensation. In this phase, the expression of cell–cell adhesion (*N-Cam*) and cell–ECM (*Tenascin C, alpha 5 integrin*) genes is increased, as is that of *Activin* βA and other transcription factors. The third period, named the precartilage period, is characterized by the upregulation of genes expressed in the previous period in addition to other genes, such as ECM genes and transcription factors (**Figure 4A**). In particular, the expression of *Sox9* is several times higher in this stage than in the previous periods. It is possible that this increase allows the commencement of molecular network control to regulate the expression of genes involved in the establishment of the cartilage phenotype (Lorda-Diez et al., 2011).

Altogether, *Sox9* is very important for the control of cell fate decisions, i.e., the commitment of mesenchymal stem/precursor cells to the cartilage lineage. As mentioned above, cartilage differentiation continues after *Sox9* is expressed in mesenchymal cells (**Figure 4B**), promoting cell aggregation and the expression of cartilage-specific proteins such as type II collagen, aggrecan, and sulphated proteoglycans. Additionally, in the absence of *Sox9* or after deletion of its expression in prechondrogenic mesenchyme, cartilage differentiation is inhibited, preventing the formation of skeletal elements. Massive cell death rather than cartilage differentiation is observed in *Sox9* mutant mice, suggesting that undifferentiated mesenchymal cells are committed to differentiation or to cell death depending on their positions inside the limb (Akiyama et al., 2002). Misexpression of *Sox9* induces extra digit formation in developing limb buds and attenuates hypodactyly caused by a *Hoxa13* mutation (Akiyama et al., 2007). *Sox9* expression in mesenchymal progenitors precedes chondrogenesis and increases at the onset of *Sox5* and *Sox6* expression in early chondrocytes (Lefebvre, 2019).

In summary, the programme of cartilage differentiation starts once *Sox9* is expressed and then cell aggregations are established to preconfigure skeletal elements; these aggregations give rise to skeletal elements shaped by proliferating chondrocytes. The early chondrocytes proliferate and secrete ECM composed mainly of collagen type II, aggrecan, and link protein. During the early stages of precursor mesenchymal cell condensation, vascular regression occurs in the mesenchyme of developing limbs (**Figure 4B**; Hallmann et al., 1987). This vascular regression induces reductions in oxygen levels. Under such hypoxic microenvironments, *Hif1a* is expressed. *Hif1a* expression is considered an important factor promoting the expression of *Sox9* and other factors that enable chondrocytes to adapt to hypoxic conditions (Amarilio et al., 2007). Furthermore, expression of Sox9 in precartilage condensations also regulates angiogenic patterning (Eshkar-Oren et al., 2009). The expression of the chondrogenic SOX trio genes (*Sox9, Sox5,* and *Sox6*) controls cell cycle progression in the proliferating chondrocytes of the central regions of cartilage elements (Akiyama et al., 2002). Thus, the expression of *Sox9*, *Sox5*, and *Sox6* are implicated in the maintenance of the cartilage phenotype. In contrast, Dy et al. demonstrated that Sox*9* is also required to activate cartilage hypertrophy (Dy et al., 2012).

The orchestrated regulation of the molecular network that controls the differentiation of cartilage leads to the establishment of embryonic skeletal elements, subsequently initiating the maturation process leading to cartilage hypertrophy and thus to elongation of the skeletal elements.

### Establishment of the Synovial Joints

Functional joints require the coordinated expression of many molecules that participate in the joint commitment and differentiation of cartilage, bone, meniscus, and other tissues. During normal limb development, the process of joint formation occurs concomitantly with the formation of the cartilage anlagen. Diarthrodial or synovial joints, such as the phalange, knee, and elbow joints, result from segmentation of continuous skeletal elements into individualized skeletal structures.

**Figure 5:**
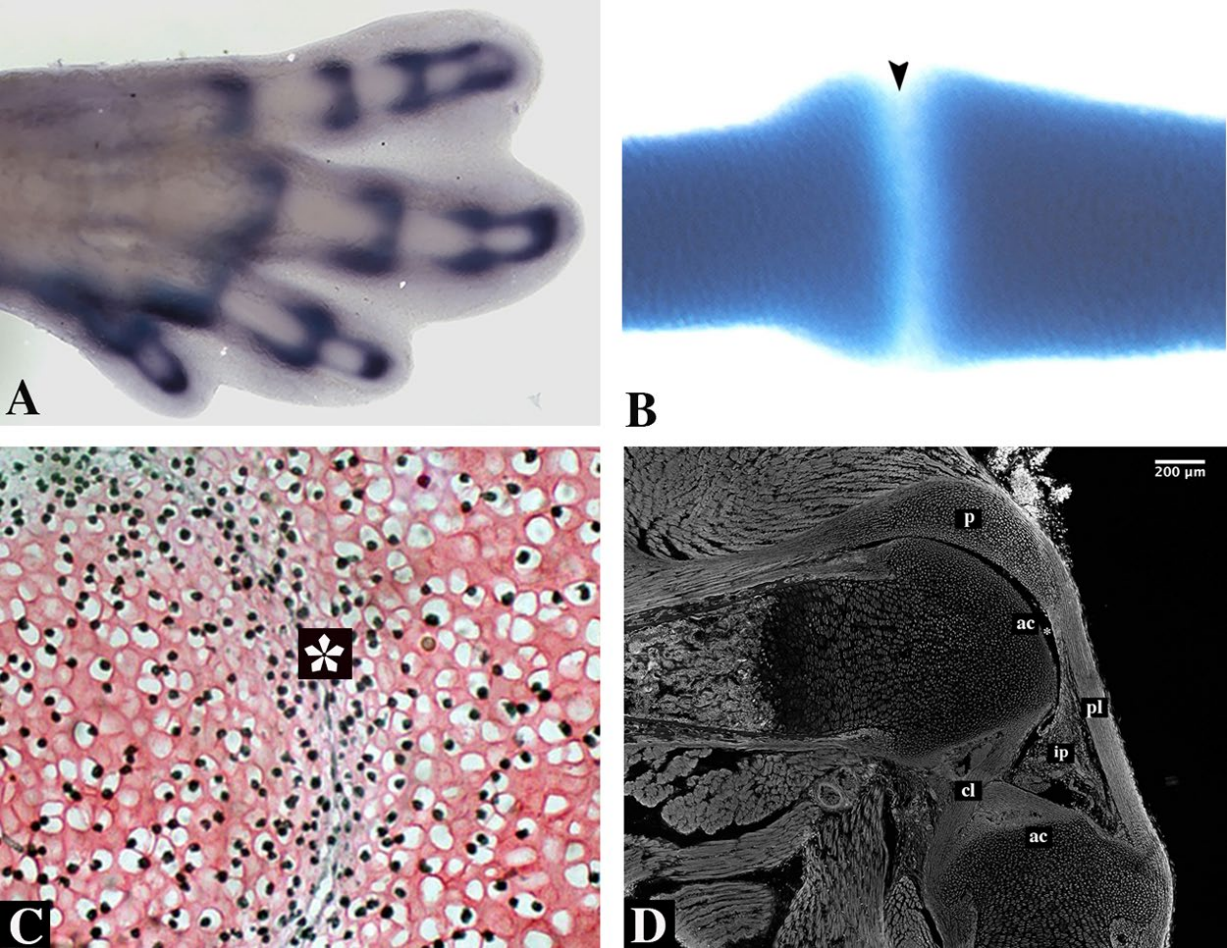
Synovial joint formation. **(A)** 31HH chick hindlimb showing an in situ hybridization for Gdf5. **(B)** Alcian blue staining of a developing digit of a turtle embryo. Notice lack of staining in the interphalangeal zone (arrowhead). **(C)** An early developing joint stained with Safranin O of a E13.5 mouse embryo showing the interzone (asterisk). Subpopulations from this area give rise to the different tissues of the joint. **(D)** A sagittal section of a knee-joint of a newborn mouse stained with phalloidin showing the different tissues of the joint; articular cartilage (ac), cruciate ligaments (cl), cells that will give rise to the infrapatellar pad (ip), patella (p), and patellar ligament (pl). Joint cavity is indicated with an asterisk.

The first evidence of joint development is the formation of the interzone (IZ), a specific region of higher cell density that forms where chondrocytes flatten and become fibroblastic tissue (**Figure 5**). During the establishment of the IZ, differentiation of the articular cartilage occurs, and separation of the two skeletal elements later occurs by a process named cavitation, thereby forming the joint capsule. It has been demonstrated that the most important process for joint establishment is the formation of the IZ. Holder (1977)and later Rux et al. (2019) found that joint formation is inhibited after removal of the IZ, resulting in a lack of segmented skeletal elements.

The formation of the IZ begins at the site of the future joint, establishing the boundary between two prospective individual skeletal elements. The flattened, fibroblastic-like cells of the IZ evolve into three cellular layers: one central intermediate lamina with low cell density flanked by two areas of high cell density. The two areas of high density differentiate into articular cartilage covering the articulating surfaces at either end of the future joint. In the case of the future knee, the central layer gives rise to the internal elements of the joint: the synovial tissue, menisci, and joint ligaments. The joint capsule arises from the mesenchymal sheath surrounding the entire IZ (**Figure 5**; Rux et al., 2019). In addition, many studies have been demonstrated that movement and mechanical forces are important to joint development and its maintenance (Drachman and Sokoloff, 1966; Kahn et al., 2009; Felsenthal and Zelzer, 2017).

#### Molecular Scenario to Trigger Joint Development

Each mature skeletal element is organized with a diaphysis in the central portion and an epiphysis at each end. The epiphysis is the region resulting from joint formation. The newly formed individual embryonic skeletal element continues to grow; remodelling of the cartilaginous template into bone occurs in the diaphysis, in which proliferating chondrocytes become pre-hypertrophic and then hypertrophic, initiating the process of endochondral ossification.

Thus, a pivotal process in joint induction is the signalling of proliferating chondrocytes to begin the programme of joint formation while stopping the programme of cartilage differentiation, preventing endochondral ossification (Garciadiego-Cazares et al., 2004). At the molecular level, cells of joint IZs begin to express different members of the Wnt gene family, such as Wnt4, Wnt9a, and Wnt16 (Guo et al., 2004). Signalling by Wnt9a and Wnt16 involves the activation of β-catenin. *Wnt9a* and β-catenin misexpression trigger a molecular cascade of joint marker expression at chondrogenic sites but may not induce true ectopic joints (Hartmann and Tabin, 2001; Guo et al., 2004). In mice lacking β-catenin in the entire limb mesenchyme the onset of *Gdf5* expression and other joint markers is not affected. Yet, no true joints are formed as *type II collagen* expression is not down-regulated (Spater et al., 2006a). Similarly, joint fusion defects are observed upon deletion of β-catenin in *type II collagen* expressing cells (Guo et al., 2004). Furthermore, joint initiation is not affected by the single or combined loss of two of the Wnt ligands, Wnt9a and Wnt4, expressed in the joint interzone, yet, their loss leads to ectopic chondrocyte differentiation in joint structures (Spater et al., 2006b). Together, these observations suggest that Wnt/β-catenin signalling is not necessary for the initiation of the joints but for their subsequent maintenance. Kan and Tabin (2013) demonstrated that c-Jun is expressed in prospective joints in a similar manner as Wnt9a and Gdf5. Interestingly, the active form of c-Jun is observed during joint development, and its expression and activity coincide with Wnt9a expression in the IZ region. In addition, c-Jun binds directly to the Wnt9a promoter, activating its expression. In mice lacking c-Jun in the mesenchyme of the early limb bud, mainly interphalangeal joint initiation and successive programmes of joint differentiation are inhibited, although other joints are also affected. The formation of many synovial joints in the appendicular skeleton is inhibited because the joint IZ is disrupted; it has also been found that c-Jun is involved in postnatal joint maturation. Inhibition of the expression of Wnt9a/Wnt16 results in continuous skeletal elements that never form an IZ. However, it is important to establish that although Wnt/β-catenin signalling occurs very early in the joint IZ, it is not necessary for early joint formation; moreover, it is regulated downstream by c-Jun. Therefore, it is possible to speculate the existence of a process of de-differentiation of proliferating chondrocytes that, if controlled by Wnt/β-catenin, is subordinate to IZ specification. In addition, there is no evidence regarding whether these de-differentiated cells are joint stem cells. Although c-Jun induces commitment according to the molecular cascade of joint formation, it is possible that some proliferating chondrocytes are converted into joint stem cells through c-Jun activation (Kan and Tabin, 2013).

While Wnt9a commits the joint IZ, Sox9 expression continues in proliferating chondrocytes, but matrillin-1 expression is inhibited (Hyde et al., 2007). Therefore, it is possible that Wnt/β-catenin signalling, once activated by c-Jun, regulates the continued differentiation of chondrocytes to prevent endochondral ossification promoting joint formation. It was mentioned above that Sox9 physically interacts with β-catenin during cartilage development. Thus, it is probable that during joint IZ formation, the ratio of Sox9 to β-catenin may lead to the induction of the molecular cascade to prevent chondrocytes from undergoing endochondral ossification. However, the effects of c-Jun regarding joint formation are restricted to interphalangeal joint initiation. Similar results have been found in the mice lacking the TGF-β type II receptor gene (TGF*β*; Spagnoli et al., 2007).

The molecular cascade of joint formation indicates that members of the BMP family, such as Gdf5, Gdf6, Bmp7, and Bmp2 (Francis-West et al., 1999a; Merino et al., 1999a; Francis-West et al., 1999b; Storm and Kingsley, 1999) are expressed in the IZ. Overexpression of BMPs expands the cartilage differentiation zone and inhibits joint formation (Duprez et al., 1996; Brunet et al., 1998; Storm and Kingsley, 1999), while, loss of noggin affects all joints (Brunet et al., 1998; Tylzanowski et al., 2006). In contrast, single mutations in humans or single or double KO of Gdf5 or Gdf6 in mice results in joint fusion (Settle et al., 2003). However, despite the specific expression of BMP family members in developing joints, these molecules are not involved in the induction of the IZ or in the formation of ectopic joints. On this basis, many studies have focused on determining the progeny of IZ cells. Using the reporter mouse line Gdf5-Cre;R26R-LacZ to map the fates of these cells and other studies have determined that the joint IZ is formed by Sox9+ and Matrillin-1, Gdf5+ cells and that they are present in the articular cartilage, meniscus, and synovial lining. These results might suggest that Gdf5+-descendent cells from the IZ become different progenitor cells that will differentiate into the tissues that compose the mature joint, such as the articular cartilage, meniscus, synovial lining, and joint capsule (Koyama et al., 2008).

However, a recent study (Shwartz et al., 2016) suggests a distinct model in which there is a constant inflow of new GDF5+ cells that are recruited into different joint cell lineages, demonstrating that cells from various joint tissues originate from GDF5+ interzone cells. In the knee, GDF5+ cells are observed in the epiphysis, articular cartilage, meniscus, and intra-articular ligaments, and they are recruited at different developmental stages of joint formation. The elbow and metacarpophalangeal joints show a similar trend, but they differ in the time in which GDF5+ cells contribute to specific tissues. Thus, joint development involves constant recruitment of new Gdf5-positive cells derived from Sox9+ chondroprogenitors (Sox9+/GDF5-), and they must presumably express *Sox5* and *Sox6*, since the deletion of both genes in chondrocytes results in a joint morphogenesis block, but if *Sox5* and *Sox6* inactivation occurs in GDF5+ cells, it results in milder joint defects and normal growth plates (Dy et al., 2010). In addition, it has been found that Tgfbr2+ cells are present within specific niches throughout embryonic life to early adulthood. They express markers of joint progenitors such as Gfg5 and Noggin (Li et al., 2013). However, it is unknown if they represent cells that originate from or give rise to Sox9+ Gdf5- chondroprogenitors.

On the other hand, it has been proposed in a previous study that whether proliferating chondrocytes enter the joint formation, or the endochondral ossification programme depends on the presence or absence of α5β1 integrin (Garciadiego-Cazares et al., 2004). In that work, it was shown that cell–ECM interactions are important during joint formation and cartilage differentiation. The study demonstrated that inhibition of α5β1 integrin signalling was necessary for joint formation. Inhibition of the function of α5 or β1 integrin with specific neutralizing antibodies or arginine-glycine-aspartic acid (RGD)-blocking peptides in forelimb organ cultures resulted in the formation of an ectopic joint. This was a consequence of inhibition of endochondral ossification. During the formation of ectopic joints, the molecular cascade of joint formation is triggered by the disruption of proliferating chondrocyte–ECM interactions mediated by α5β1 integrin. Wnt9a, Gdf5, chordin, autotaxin, type I collagen, and CD44 are expressed in ectopic joints, whereas Indian hedgehog (IHH) and type II collagen are downregulated. As mentioned above, BMP signalling potentiates chondrogenesis and inhibits joint formation, but after inhibition of α5β1 integrin, BMP signalling improves joint formation (Garciadiego-Cazares et al., 2004). These data strongly suggest that once the molecular cascade of joint formation is initiated, BMP signalling contributes to joint formation. As mentioned by Attila Aszodi and his group (Docheva et al., 2014), “although this hypothesis is clearly attractive, genetic experiments with conditional knockout mice did not support a mechanistic role α5β1-mediated matrix attachments in joint morphogenesis”. However, the β1 integrin allele was floxed under the Col2a1-cre or Prx1-cre promoter in transgenic mice. Thus, an inducible β1 integrin or α5 integrin allele under the Col2a1-cre promoter may give a better response.

In conclusion, the formation of the IZ is an excellent model with which to study the mechanisms that control the appearance of joint stem/progenitor cells during development. Until now, it has remained unknown if the IZ appears and then the joint stem/progenitor cells migrate or dedifferentiate from other sources or if the stem/progenitor joint cells determine IZ formation. It is possible that new microenvironments for IZ formation are created during the establishment of the joint IZ within a source/reservoir of joint stem/progenitor cells that will ultimately form the distinct tissues of mature joints, such as the articular cartilage, meniscus, synovial lining, and joint capsule.

### Stem Cell Renewal and Cell Differentiation During Endochondral Ossification

**Figure 6:**
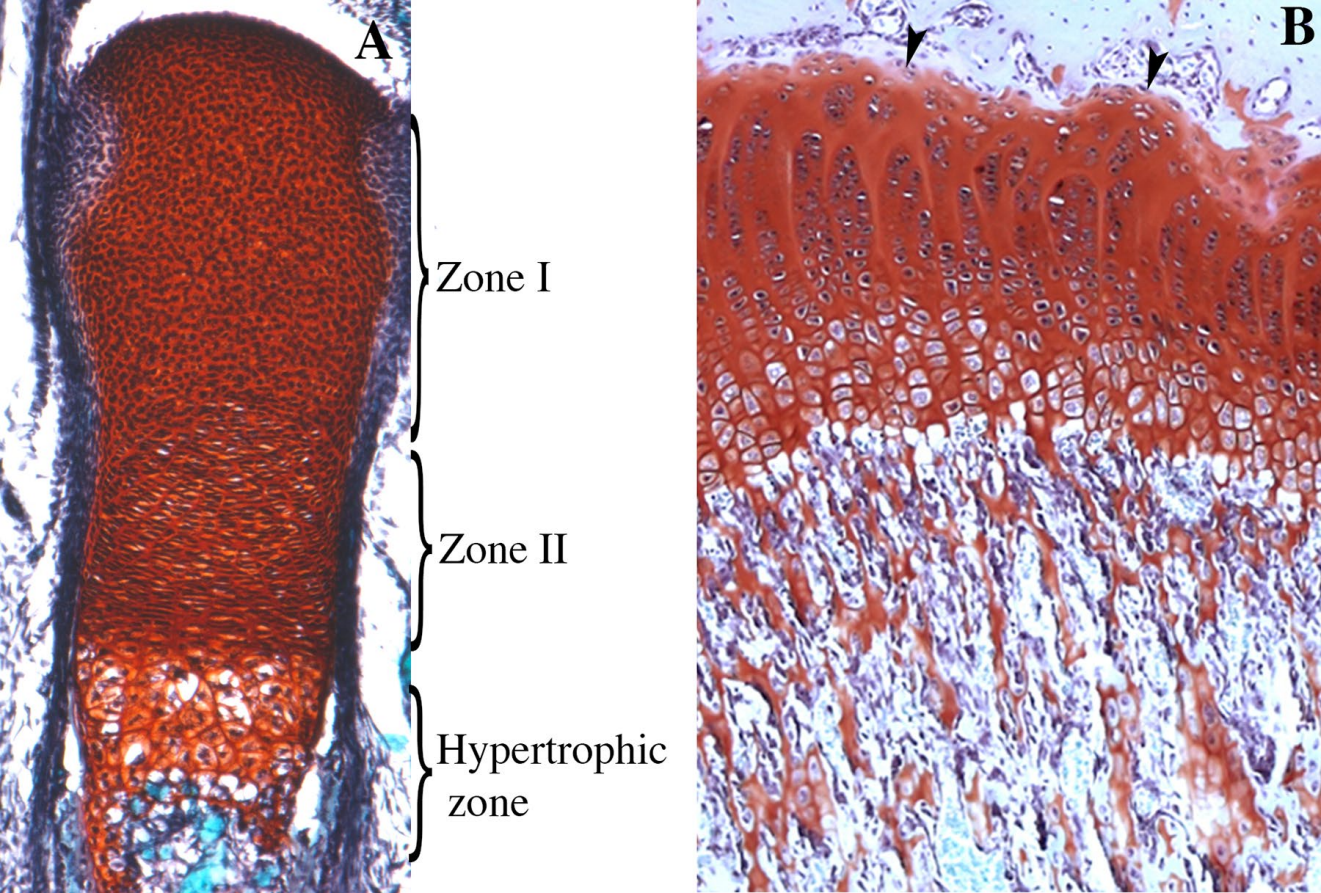
Skeletal stem cells during endochondral ossification. **(A)** Longitudinal section of an E18.5 mouse tibia stained with Safranin O. Zone I, zone II, and the zone of hypertrophic chondrocytes are represented. Zone I corresponds to the entire epiphysis and contains differentiated chondrocytes that in this stage are all proliferative and originates the articular cartilage. Zone II corresponds to cells, originated from zone I, organized in parallel columns. **(B)** Growth plate of 6-weeks-old rat. In the juvenile stage, chondrocytes from the resting zone (arrowheads) behave as stem cells giving rise to different clones disposed in a column, that contributes to the longitudinal growth of the skeletal elements. In foetal, neonatal, and juvenile stages columns extend up to the primary spongiosa (ps).

Endochondral ossification programme initiates in the centres of the skeletal elements. This process is characterized by the replacement of cartilaginous templates with bone. In the peripheries of the skeletal elements, flattened cells differentiate into perichondrial cells. Proliferative chondrocytes differentiate into permanent articular cartilage at the end of the skeletal elements giving rise to zone I chondrocytes that are round or slow-proliferative. In the centres of long bones are the proliferating chondrocytes or Zone II chondrocytes, which exhibit flat morphology and are organized in parallel columns. At the centres of skeletal elements, proliferating chondrocytes undergo differentiation to form first prehypertrophic and then hypertrophic chondrocytes. Surrounding the newly formed skeletal elements, there is a thin layer of mesenchymal cells that will form the perichondrium; when ossification begins, this layer will form the periosteum (**Figure 6**). During this process, environmental conditions change to support vascular invasion, and periosteal cells invade the zone of hypertrophic cells to begin bone formation (Kozhemyakina et al., 2015). The same process of endochondral ossification is recapitulated during the formation of growth plates in infants. These structures are formed through secondary ossification, so they are localized between the primary ossification centre and the newly formed ossification centre (**Figure 6**). Notably, the proliferative activity of resting chondrocytes in the growth plates of bones during embryonic and foetal development is higher than that during puberty. Then, proliferating chondrocytes become prehypertrophic and undergo hypertrophy and mineralization. This coordinated process enables longitudinal growth in both embryonic and postnatal skeletal elements (Karsenty and Wagner, 2002; Kozhemyakina et al., 2015).

#### Early Endochondral Ossification: From Proliferative to Hypertrophic Chondrocytes

A pivotal point of control for the growth of skeletal elements is the transition of resting chondrocytes into proliferating chondrocytes followed by the differentiation of these cells into hypertrophic chondrocytes. The mechanism by which signalling molecules control cell proliferation and differentiation involves key intracellular regulatory factors, including cell cycle regulators and transcription factors. Proliferating chondrocytes exit the cell cycle through the activity of the cyclin-dependent kinase (CDK) inhibitors p21, p57^Kip2^, p107, and p130 (Rossi et al., 2002; MacLean et al., 2004; Goldring and Marcu, 2009). Similar to the case during the onset of cartilage differentiation, the proliferating chondrocytes still express Sox9, which together with Sox-5 and Sox-6 maintain the cartilage in a proliferative stage (Ikeda et al., 2004; Kim et al., 2011). This transcription factor regulates the expression of ECM cartilage genes such as *collagens* (*types 2, 9,* and *11*) and proteoglycans (*aggrecan*) (Bi et al., 1999). Hypertrophic differentiation depends on *Runx*-2, which directs *collagen 10a1* (*Col10a1*) and matrix metalloproteinase 13 (*Mmp13*) expression (Takeda et al., 2001). It is well known that during this process, Runx-2 inhibits Sox-9 function, and *Col2a1* and *aggrecan* are consequently downregulated (Zhou et al., 2006). In contrast, conditional *Sox9* or Sox trio (*Sox9, Sox5*, and *Sox6*) overexpression delays or inhibits hypertrophic differentiation (Ikeda et al., 2004; Kim et al., 2011), respectively.

With regard to the transition from chondrocyte proliferation to differentiation, two signalling molecules, parathyroid-related protein (PthrP) and IHH, interact in a negative feedback loop to regulate the onset of hypertrophic differentiation (Vortkamp et al., 1996). *PthrP* is expressed in the perichondrium, while its receptor, *Ppr*, is expressed in proliferative chondrocytes (Amizuka et al., 1994; Karaplis et al., 1994) and promotes chondrocyte proliferation (Lanske et al., 1996). IHH is expressed in prehypertrophic chondrocytes and binds to its receptor Patched, which is also expressed in chondrocytes and perichondral cells (Tavella et al., 2004). Activation of the IHH pathway induces *PthrP* expression in perichondral cells, promoting the proliferation of chondrocytes (Vortkamp et al., 1996). Cartilage hypertrophic differentiation begins when the proliferating chondrocytes are sufficiently far from the source of PTHrP. The cells stop proliferating and, then the site at which chondrocytes differentiate into hypertrophic cartilage is established. Then, IHH is expressed together with type X collagen, which is characteristic of hypertrophic cartilage. However, IHH is only expressed in early hypertrophic chondrocytes. In this process, other signalling molecules are involved, such as Wnt, FGF, and BMP (Kozhemyakina et al., 2015).

#### The Growth Plate Formation

As the first ossification centre enlarges, the secondary ossification centre is established in the epiphyses (proximal or distal) of long bones. Here, chondrocytes stop proliferation and undergo hypertrophy, attracting blood vessel invasion. Between primary and secondary ossification centres, growth chondrocytes continue to proliferate, forming a distinct plate of cells known as the growth plate. At the top of the growth plate, the proliferation rate of round chondrocytes slows, and these cells are then referred to as resting chondrocytes (Kronenberg, 2003). In humans, secondary ossification centres form during late embryogenesis, while in the mouse they are formed during early postnatal development P7–P15. In these structures, cell differentiation is active and is responsible for the growth of long bones, while in the articular cartilage, the rates of proliferation and differentiation are severely reduced (Kozhemyakina et al., 2015).

During foetal and neonatal endochondral ossification, resting chondrocytes with rounded morphology behave like stem cells, while proliferating chondrocytes behave like transient amplifying cells. Research using clonal genetic tracing with multicolour reporters or *Pthrp-mCherry* knock-in reporter alleles has demonstrated that cells of the resting zone give rise to flat chondrocytes of the proliferating zone and to hypertrophic chondrocytes by clonogenic division (Mizuhashi et al., 2018; Newton et al., 2019). The findings suggest that the cells of the resting zone give rise to all the cells clonogenically. However, the cells in the resting zone become depleted, and bone growth in the first ossification centre does not continue. Then, the supply of proliferating and hypertrophic chondrocytes ceases. Once the secondary ossification centre begins to form, the growth plate is established. Here, among resting chondrocytes, some *Pthrp-mCherry* cells behave like stem cells. They acquire the capacity for self-renewal, resulting in the formation of entire monoclonal columns of chondrocytes from the resting zone to the hypertrophic zone. In these niches, the skeletal stem cells begin to express stem cell markers and to undergo symmetric cell division, thus renewing the population of stem cells (Mizuhashi et al., 2018; Newton et al., 2019).

The control of both self-renewal and cell differentiation of skeletal stem cells is important for maintaining the pool of these cells. These processes are associated with determination of stem cell proliferation and stem cell identity. Hedgehog signalling and Pthrp signalling are both involved in promoting self-renewal and cell proliferation. *Pthrp-mCherry* cells are less proliferative than proliferating chondrocytes and are present in the growth plate in the CD45neg cell population (Chan et al., 2018; Mizuhashi et al., 2018). In the growth plate, activation of the IHH pathway induces *PthrP* expression in resting cells. Inhibition of hedgehog signalling reduces the clonal size of chondrocytes in the resting region but does not cause premature hypertrophy of the cartilage. This suggests that IHH controls the proliferation of skeletal stem cells (Newton et al., 2019). On the other hand, it has been reported that activation of mTORC1 (Newton et al., 2018) controls the organization of the resting zone without affecting cell proliferation. Activation of this signalling pathway regulates stem cell renewal because it controls the transition of skeletal stem cell division from asymmetric to symmetric.

### Concluding Remarks

Our understanding of skeletal development has grown considerably in recent years. From the perspective of developmental biology, we have deep knowledge of the molecular mechanisms that control the shaping and positioning of cartilage and bone primordia during embryonic development. The use of genetic and molecular approaches together with experimental manipulation of embryos *in vivo* has favoured exploration of the requirements for particular genes at early stages during the commitment of mesenchymal cells. Such approaches have also allowed researchers to elucidate how committed cells are organized in space and time to produce three-dimensional skeletal organization and to drive morphogenesis and patterning. This knowledge is of great interest in disciplines such as the study of skeletal malformations and particularly in newly emerging fields such as regenerative medicine. However, this progress presents new challenges, such as how to use this information to rebuild damaged tissues. The rapid expansion of stem cell research has allowed us to understand how stem cells acquire different cell fates during development and how these cells can contribute to the formation of damaged tissues or regenerate them *in vitro*. Knowledge of stem cells provides indications/clues about how to activate the endogenous programme of cell differentiation to a correct fate and the creation of organoids in non-regenerative tissues. On the other hand, progress in the design of appropriate materials for controlling cell fate behaviour and or mimicking the ECM has generated advances in regenerative medicine but has not necessarily achieved the recapitulation of *in vivo* development. This is especially important in transplantation because the response of the newly delivered cells to their new environment is usually unknown. Complications with currently used materials, the control of factor release kinetics, and the vascularization and innervation of the tissue to allow graft survival and function persist. To promote the tissue regeneration of a whole limb (or any other limb tissue) *via* regenerative medicine, it will first be necessary to obtain a deeper understanding of the cellular mechanisms that regulate the microenvironment, the expression of morphogens, and the activation of signalling pathways in appropriate sites and cells, without ignoring how and what types of signals regulate the early cell fate decisions of the stem/progenitor limb cells. Thus, to repair or regenerate a tissue or structure in a whole limb, the translation of basic research discoveries to clinical applications must be considered.

Concerning developmental biology, knowledge regarding stem/progenitor cells and their interactions with their microenvironment *in vivo* is pivotal to understanding how mesenchymal cells sequentially undergo developmental commitment during skeletogenesis, transforming from mesenchymal cells to cartilage/bone cells; from cartilage cells to joint cells; from joint cells to articular cartilage, meniscus, synovial lining, and joint capsule cells; and from cartilage cells to bone cells during endochondral ossification. A main question associated with the study of skeletal development is whether all skeletal stem cells originate from undifferentiated mesenchymal cells at early stages of limb development. If not, where do these stem cells come from, or how are they locally induced? Are the joint IZ regions reservoirs of stem cells or progenitor cells? Is IZ formation a consequence of local induction of joint stem cells? Further studies are needed to find new skeletal stem cell niches for the various developing tissues during skeletogenesis.

## Author Contributions

JCM-L and JC-M conceived and wrote the paper. DG-C wrote the paper.

## Funding

This work was supported by grants to JC-M (IN211117 DGAPA-PAPIIT-UNAM, 168642 CONACYT and 1887 Fronteras de la Ciencia 2018, CONACYT). JCM-L received a postdoctoral fellowship (28971) from Fronteras de la Ciencia 2018, CONACYT.

## Conflict of Interest

The authors declare that the research was conducted in the absence of any commercial or financial relationships that could be construed as a potential conflict of interest.
